# A three-armed randomised controlled trial investigating the comparative impact of guidance on the efficacy of a web-based stress management intervention and health impairing and promoting mechanisms of prevention

**DOI:** 10.1186/s12889-021-11504-2

**Published:** 2021-08-05

**Authors:** Patricia Nixon, Leif Boß, Elena Heber, David Daniel Ebert, Dirk Lehr

**Affiliations:** 1 Department of Health Psychology and Applied Biological Psychology, Institute of Psychology, Leuphana University of Lueneburg, Lueneburg, Germany; 2 Department for Sport & Health Sciences, Technical University of Munich, Psychology & Digital Mental Health Care, Munich, Germany; 3 GET.ON Institute for Online Health Trainings, Hamburg, Germany

**Keywords:** Web-based, Occupational health, Stress management, Randomised controlled trial

## Abstract

**Background:**

Web-based stress management interventions (SMI) fit increasingly digital lifestyles, reduce barriers of uptake and are easily scalable. SMIs might lower levels of stress in employees and thereby contribute to the prevention of depressive symptomatology. Different guidance formats can impact the efficacy of SMIs, with higher intensity assumed to result in larger effects. However, head-to-head comparisons of guidance formats are rare. This is the first trial to examine the impact of adherence-focused guidance compared to self-help on the efficacy of an occupational SMI compared to a wait list control condition. Additionally, it will be investigated if the SMI enfolds its impact on preventing depressive symptomatology by different pathways through reducing health impairing and increasing promoting factors.

**Methods:**

A three-armed randomised controlled trial (RCT) on an occupational SMI was conducted. 404 employees with elevated levels of perceived stress (PSS-10 ≥ 22) were randomly assigned to: adherence-focused guidance (AFG), self-help (SH) or a wait list control group (WLC). The primary outcome was perceived stress (PSS-10). Secondary outcomes included health- and work-related measures. A parallel mediation analysis with stress and resilience as mediators for the effect on depression (CES-D) was carried out. Data collection took place at baseline (T1), after 7 weeks (T2) and 6 months (T3).

**Results:**

The SMI was effective for all groups on the primary and secondary outcomes. For stress, analyses of covariance (ANCOVA) revealed significant group effects at T2 (F_2,400_ = 36.08, *P* < .001) and T3 (F_2,400_ = 37.04, *P* < .001) with large effect sizes for AFG (T2: d = 0.83; T3: d = 0.85) and SH (T2: d = 0.88; T3: d = 0.91) compared to WLC. No significant group differences were found for the efficacy between AFG and SH on the outcomes. Adherence in terms of completed modules was significantly higher for AFG compared to SH. The SMI’s impact on depression was mediated by perceived stress: a_1_b_1_ = − 0.77, 95% CI [− 1.26, − 0.34] and resilience: a_2_b_2_ = − 0.62, 95% CI [− 1.05, − 0.26].

**Conclusions:**

The SMI was effective for reducing stress and improving other health- and work-related outcomes, irrespective of the guidance format. Results did not demonstrate superiority of adherence-focused guidance for the efficacy but for adherence in terms of completed modules. Among other reasons, better communication strategies about offered guidance and awareness-raising measures are discussed. Results from mediation analysis suggest that preventive SMIs should be designed to reach two goals: reducing the risk factor of stress and simultaneously increasing health promoting factors such as resilience.

**Trial registration:**

German Clinical Trial Registration (DRKS) DRKS00005687, 6/6/2014.

## Background

Work-related stress is associated with increased risk for development and maintenance of adverse physical [1–3] and mental health consequences [4]. Experiencing stressors at work is associated with an elevated risk of sickness absence due to a diagnosed mental disorder [5] and can precede depressive symptoms over time, thereby increasing the risk for subsequent depression [6, 7]. As one of the three leading causes, unipolar depression largely contributes to the burden of disease and is predicted to be number one by 2030 [8]. The World Health Organization (WHO) therefore calls for prevention to effectively reduce this tremendous burden [9].

This in turn leads to substantial socio-economic harms. These can be divided into direct costs due to growing use of health care services and indirect costs resulting from productivity loss caused by work loss days, presenteeism, employee turnover and work impairment [10, 11]. Notably, indirect costs account for most of the total stress-related cost with 70–90% [10]. As a national example, work stress, depression and anxiety predominantly caused for 44% of ill health cases and 57% of lost working days in Great Britain in 2017/2018 [12].

Ongoing digitisation and rapidly developing technology altered the labour market profoundly with a transformation towards the Fourth Industrial Revolution. As a result, employees can experience severe stress due to unfavourable changes of workplace cultures, employee turnover or job insecurity [3, 13, 14]. The other side of the coin is the potential of using digital facilities to deliver web-based interventions which have been shown to be effective in reducing stress [15] and improving mental health in those affected by serious disorders such as depression [16, 17]. Not least, the COVID-19 pandemic has highlighted the need for the implementation of e-health interventions into routine health care and overcoming barriers to effectively do so [18].

From a public health point of view, Internet Interventions generally may offer many advantages compared to face-to-face approaches. They fit increasingly digital lifestyles, are easily scalable and reduce barriers of uptake [19]. Furthermore, health economic evaluations showed that a guided version of the utilised SMI from prior studies was cost-effective [20, 21].

Reaching a greater proportion of affected individuals that otherwise may remain untreated hence seems more feasible [22]. Substantial benefits of this delivery mode are the portability and time-spatial independent accessibility [23] allowing users to review contents at their own pace. The anonymity of the Internet might circumvent fears of possible stigmatisation or self-disclosure in traditional therapy settings that for such reasons may be rejected by many subjects in need [24, 25]. Supporting evidence stems from studies showing that the web-only delivery mode can be as effective as face-to-face settings [26–28].

Web-based occupational stress management interventions (SMI) seem to be a promising approach against this background. Studies have demonstrated treatment effects on various health outcomes within the occupational context [29] with maintaining reductions of stress for up to 6 months [15, 30]. Meta-analytic evidence showed that exposure to work-related stressors could precede the onset of depressive symptoms [31] and clinical depression [6]. There are three well investigated and prominent theories and models to explain these associations, namely the demand-control model, effort-reward imbalance model and organisational injustice framework [32]. There is evidence that each of these theoretical approaches is valid and correctly predicts that stressors at work are linked to a moderately elevated risk of the onset of depression [32]. Against this background, effects of SMI on depression were also investigated and found to be positive at post-intervention and at 6-month follow-up, although larger for the former [15, 30, 33, 34]. Notably, these studies are characterised by moderate to high heterogeneity [15, 30].

A fundamental aspect varying along these studies that seems to be crucial in affecting the efficacy of SMI, is the impact of human guidance [35]. The intensity of guidance can differ regarding the format and scope of provided human support. On the one side, intensive guidance is expert driven, requiring a high invest of resources. It can for example be content-focused with personalised written feedback on completed exercises or modules [15]. On the other side and opposed to this format, a self-help format requires only few resources and provides no professional support [36]. This format can easily be scaled to maximum, is cost-effective and needed to sufficiently treat subjects who otherwise would not receive any professional service at all [20, 37]. Adherence-focused guidance is an attempt to find a good compromise between those two intensities in guidance. This guidance format was established in earlier studies and consists of adherence monitoring and feedback on demand [35, 38]. Therefore, adherence-focused guidance is client driven, requiring an active role and initiative of a participant when in need of support. Providing guidance by human support is expected to be conducive to the efficacy of Internet Interventions [15, 39, 40]. The support provided usually is of a technical or clinical kind or focusses on the correct usage of the intervention [41]. According to the Supportive Accountability Model [42], human support enhances adherence to e-health interventions. Social presence or performance monitoring by an e-coach that is perceived as benevolent and trustworthy are for example integral factors to foster accountability. Studies demonstrated declining clinical outcomes with decreasing coaching time spent on each participant, with an increasing likelihood of study dropout at the same time [38, 43, 44]. Further findings indicate that guidance also seems to impact user satisfaction and acceptance that both in turn affect adherence [45–47]. Previous trials suggested the use of adherence-focused guidance [48] and showed that it was effective for Internet Interventions targeting stress management [49] or subthreshold depression [50]. Investigating different guidance formats is particularly important since little or no adherence and low uptake rates are major issues of Internet Interventions [45, 51]. Until now, there is no evidence on the comparison of adherence-focused guidance to self-administered formats for SMI unguided interventions, although empirical validations of the effects of varying guidance formats are highly necessary for large-scale dissemination into routine health care. Against this background, this study aims at comparing the effectiveness for adherence-focused guidance which combines adherence-monitoring and human support, with a self-help mode.

While guidance is crucial for an evidence-based design of SMI, the underlying mechanisms of how such interventions come into effect have not been fully captured yet. Despite an increasing number of studies, the process by which SMI result in effects on depression is unclear. A theoretical framework that can be applied to address this gap is the job-demands-resources (JD-R) model that was developed to explain the genesis of job strain [52, 53]. According to this model, any occupational characteristics can be classified into one of the two underlying clusters: job demands or job resources. These two clusters initiate two diverging processes. Job demands that no longer can be met instigate a health impairment process, whereas the availability of sufficient job resources launches a motivational process. This model can be used for this study to examine the global issue of stress in a more detailed manner. In particular, to elucidate how the SMI can affect depressive symptoms. Based on the JD-R model and earlier research on the relationships between stress and health outcomes, the SMI may work through two different paths. One of these paths can be assumed to be health impairing with work stress often being precedent to depression. Hence, positive effects on depression might be preceded by an efficacious stress reduction. Opposed to this, available resources can promote resilience that can buffer such deleterious effects and prevent pathogenesis [54, 55]. Taken together, positive effects of SMI on depression might be the result of either one of these paths acting individually or a dual pathway working simultaneously.

This is the first investigation of the comparable efficacy of different guidance formats within a single, homogenous study on SMI yet. Therefore, this three-armed randomised controlled trial has three aims. First, to assess the comparative efficacy of adherence-focused guidance, self-help and a wait list control group, assuming each of the intervention groups will be superior to the waitlist control group and adherence-focused guidance will be superior to self-help. Second, to reveal further insights into utilisation rates and acceptability of the various guidance formats. And third, to investigate mediating paths of how this SMI affects depression, considering perceived stress and resilience as potential mediators.

## Methods

### Study design and hypotheses

A three-armed randomised controlled trial (RCT) was conducted in compliance with the study protocol [35] and the Declaration of Helsinki and Good Clinical Practice (GCP). Subjects were randomised into three groups: (1) adherence-focused guidance (AFG), (2) self-help (SH) and (3) a wait list control condition (WLC). Accordingly, both AFG and SH received the same SMI, but guidance was different. Each group had full access to treatment as usual (TAU).

Based on data from an earlier pilot evaluation we expected a mean effect for the AFG condition compared to WLC of at least d = 0.70. Evidence available at the time of planning the study showed that meta-analytic data for cognitive-behavioural occupational SMI indicated an effect size of d = 0.68 [56] and that unguided Internet Interventions consistently seem to produce lower effect sizes compared to guided trainings [39]. Against this background, this study aimed to be able to detect a between group effect size of d = 0.30. To detect this effect with a power (1-ß) of 80% and α = .05 in multiple Bonferroni-adjusted tests (H1: AFG superior to WLC at post-intervention, H2: SH superior to WLC at post-intervention, H3: AFG superior to SH at post-intervention), a required sample size of 408 participants was calculated with PASS12 (NCSS).

After screening for eligibility (T0) and allocation, assessments were conducted at baseline (T1), 7 weeks post-intervention (T2) and at 6-month follow-up (T3) using a secured online-based self-report system (AES, 256-bit encrypted). Primary and secondary outcomes were used as dependent variables and treatment condition as the independent variable with respective baseline scores as covariates; for a description of the sample, baseline characteristics were assessed that are listed in the section Other measures. The University of Marburg ethics committee approved of this study (No. 2014-5 K). All participants gave informed consent. The trial was registered in the German Clinical Trials Register (DRKS00005687).

### Inclusion and exclusion criteria

Inclusion criteria required (1) participants being adults (aged ≥18), (2) current employment, (3) Internet access, (4) sufficient German reading and writing skills, (5) willingness to give informed consent and (6) scores ≥22 on the Perceived Stress Scale (PSS-10) [57]. The PSS-10 cut-off allows for the selection of subjects with elevated stress levels, as identified by one standard deviation (*SD* = 6.2) above the mean (*M* = 15.3) in a large sample of working people [58]. Exclusion criteria were previous or current diagnosis of psychosis or dissociative symptoms, or a notable suicidal ideation indicated by a score > 1 on the ninth item (“I feel I would be better off dead”) of the Beck Depression Inventory (BDI) [59].

### Recruitment and randomisation

Between January and May 2014, nationwide recruitment took place trough newsletters, press releases and the support of a large German health insurance company that advertised the study in their member journal and regional offices (*n* = 918). Participation was not limited to the health company’s insurants. Interested subjects signed up on the website (www.geton-training.de) by providing their e-mail address. After screening for eligibility, giving written informed consent and completing the baseline assessment, participants were randomly allocated to the three different intervention arms. A third independent party performed individual randomisation at a ratio of 1:1:1 and a block size of three using an automated computer-based random integer generator (DatInf® RandList). Thereafter, participants were not blinded to conditions and either received immediate access to the SMI or 6 months later if allocated to the WLC.

### Intervention

Based on Lazarus’ transactional model of stress [60], the intervention was developed for employees and focusses on problem solving [61, 62] and emotion regulation skills [63, 64]. It comprises seven core modules for psychoeducation (module 1), problem solving (modules 2–3), emotion regulation (modules 4–6), planning future (module 7) and an optional booster session offered four weeks after training completion (module 8). Table 1 supplies a more detailed description of the contents. Within modules 2–6, participants could autonomously choose to adapt their intervention for additional modules covering time management, rumination and worrying, psychological detachment from work, sleep hygiene, rhythm and regularity of sleeping habits, nutrition and exercise, organising work breaks, and social support. In that way, the intervention was tailored to the subjects’ individual needs depending on responses that they opted for. Subjects were advised to complete at least one and at maximum two modules per week. It took 45 to 60 min to complete a module, resulting in a total intervention period of approximately four to seven weeks. The interactive lessons incorporated units of various media formats, such as texts, audios and videos. Additionally, subjects could make use of an inbuilt read-out function. Further key elements were exercises, the encouragement to keep a daily stress diary and homework assignments, as the intervention aimed to assist participants in fostering newly acquired stress management techniques in their everyday life. Furthermore, participants were able to decide if they wanted to receive automatic motivational text messages and short exercises (e.g., for relaxation) to their mobile phones and if their frequency should be rather low (one text message alternately) or intense (two to three text messages daily). The responsive web application could be accessed through mobile phones and (tablet) computers. A more comprehensive description of the intervention can be found in the study protocol [35].

**Table 1:** Contents of the GET.ON stress management intervention

Module	Focus	Description
1	Psychoeducation	Introduction to the intervention and its flow, stress management basics (e.g. health impacts), individual stress analysis, stress diary, various exercises
2	Problem-solving I	Coping strategies, additional individual stress analysis, habits, problem-solving skills, various exercises
3	Problem-solving II	Advanced coping strategies and problem-solving skills, meeting challenges, various exercises
4	Emotion regulation I	Personal stressors and problems, muscle relaxation and breathing techniques, importance of regular exercising and positive health behaviours
5	Emotion regulation II	Acceptance, meaning and benefit of emotions, emotional valence, various exercises
6	Emotion regulation III	Foundations of self-criticism, self-worth, self-care, self-support in difficult times, various exercises
7	Plan for the future	Physical early warning systems, strengthening personal foundation in life, implementing intentions
8	Booster session	Reviews of previous content and exercises, realignment of goals, personal challenges, various exercises

#### Adherence-focused guidance (AFG)

Previous studies established AFG as guidance format which comprises (1) adherence monitoring and (2) feedback on demand by e-coaches [38]. Every subject was assigned to an e-coach in a one-to-one ratio throughout the intervention. The e-coaches were trained psychologists that followed guidelines for the feedback process based on the standardised manual for the intervention. Adherence was monitored by frequently checking for duly module completion and sending reminders if subjects did not finish at least one module within a week. In other studies, reminders (personal and automatic) ameliorated adherence to self-guided health promotion and health behaviour interventions [65–67]. Participants received personalised and written feedback on demand only upon request via the internal platform messaging system within 48 h. Contact to an e-coach could be established by clicking on the respective button. The provided feedback was expected to enhance adherence and therefore impact the comparative efficacy in favour of this study condition [42, 68]. Based on the Supportive Accountability Model [42], the provided human support by professional e-coaches was supposed to create perceived legitimacy and to be a requirement for positive effects of the monitoring part of AFG. Notably, it is assumed that the two elements of AFG (i.e., monitoring and feedback on demand) intertwine with each other in regard to their effects on adherence.

#### Self-help (SH)

Subjects in the SH-arm could contact the study administration and received support in case of technical issues.

#### Wait list control group (WLC)

The WLC-arm obtained access to the self-help intervention 6 months after randomisation. Priorly, they had full access to TAU through routine healthcare services.

### Measures

Data was collected online between March 2014 and July 2015 with German self-report measures at successive assessments in time: screening for eligibility (T0), at baseline (T1), 7 weeks after randomisation (T2) and at a 6-month follow-up (T3). Further questionnaires used [35] will be taken into account in future publications.

#### Primary outcome measure

Primary outcome was the subjective stress level measured by the German version of the 10-item Perceived Stress Scale (PSS-10) [57, 69]. Since the PSS-10 was developed based on Lazarus’ transactional model of stress, it was assumed to be most suitable regarding the intervention’s content. The self-report items of this well-established questionnaire assess to what extent participants experienced their lives as stressful, i.e. as overstraining, unmanageable and unforeseeable in the past month. To avoid confounding with the training period (from T1 to T2), subjects in this study were asked to answer the questions against the background of their past week [69]. Respondents evaluate the items on a five-point Likert scale from 0 (never) to 4 (very often), resulting in sum score ranges from 0 to 40. Accordingly, the higher the sum score values, the more elevated stress levels are perceived. Sample item: “How often have you felt that you were unable to control the important things in your life?”. The scale was psychometrically evaluated in a German community sample and shown to have good internal consistency and construct validity [70]. For different samples, internal reliabilities (Cronbach’s α) were found to be .78 and .91 [71].

#### Secondary outcome measures

##### Health impairing and promoting mediators

Secondary outcomes included the examination of depression severity with the short version of the Centre for Epidemiological Studies’ Depression Scale (CES-D) [72, 73], with 15 items that are responded to on a four-point Likert scale from 0 to 3 (α = .95). Sample item: “I felt depressed”. The Connor-Davidson Resilience Scale (CD-RISC) [74] was used to examine resilience, measured with ten items on a five-point Likert scale from 0 to 4 (α = .85). Sample item: “Deal with whatever comes my way”. Emotional exhaustion was measured with the corresponding subscale of the Maslach Burnout Inventory (MBI-GS-D) [75, 76]. This scale is composed of five items that are rated from 1 to 6 (α = .85). Sample item: “I feel emotionally drained from my work”.

##### Work-related health

Work engagement was examined with the Utrecht Work Engagement Scale (UWES) [77, 78] that distinguishes between the three subcomponents dedication, vigour and absorption and consists of 9 items which are assessed on a 7-point Likert scale from 0 to 6 (α = .9). Sample item: “I am enthusiastic about my job”.

##### Work-related productivity

Presenteeism was assessed with the Work Limitations Questionnaire (WLQ) [79] and a single-item work ability question (WAI) [80]. Sample items: “Experience physical or mental problems speaking to others in person/on phone”, and “How would you rate your current ability to work?” respectively. In addition, the Effort Reward Imbalance Questionnaire – Short Form (ERI-SF) [81] was used with 10 items with a Likert-scale from 1 to 4 that compose the three scales effort, reward and overcommitment (αs = .77, .82 and .83, respectively). Sample item: “I have many interruptions and disturbances while performing my job”.

##### Client satisfaction and intervention usage

The Client Satisfaction Questionnaire adapted to Internet-based interventions (CSQ-I) [82] was used to investigate the participants’ satisfaction with the intervention. This comprises eight items with a range from 0 to 4. Reported values for the reliability indicated by McDonald omegas were .93–.95. Sample item: “I received the kind of training I wanted”. For an in-depth analysis, participants were required to answer questions about the different guidance formats (e.g. “What kind of guidance would you prefer if you would participate in a training of this kind again?”, “How often did you make use of the possibility to get in touch with an e-coach?”). In order to take these figures into account, the number of inquired feedbacks, as well as the amount of messages e-coaches sent to participants was checked for on the internal platform messaging system.

##### Other measures

For a description of the sample, we assessed various demographic variables such as sex, age, marital status, ethnicity, educational level, employment, work sector of employment, income and the previous use of health services.

### Statistical analyses

The statistical analyses adhered to the pre-published study protocol [35]. They were carried out using IBM SPSS Statistics 25 [83] and according to the Consolidated Standards of Reporting Trials (CONSORT) guidelines [84, 85]. Consequently, analyses were conducted on the intention-to-treat (ITT) basis handling missing data with multiple imputations [86]. For this purpose, 10 estimates were calculated for each missing data point that were aggregated into a single overall value. To evaluate the efficacy of the intervention, analyses of covariance (ANCOVA) were conducted with respective outcome baseline values specified as covariates. Earlier simulation studies demonstrated the methodological robustness of ANCOVA for analysing experimental studies in terms of protecting against bias, higher precision and statistical power [87, 88]. For all analyses, a two-tailed significance level was set at *P* < .05. Deviating from the study protocol [35], Cohen’s *d* [89] was calculated by standardised deviations (SD) of the respective measurement points. Restricted variance in baseline scores would lead to smaller SD and the risk of an overestimation of effects. Therefore, effect sizes were calculated in a more conservative manner. Additionally, per protocol analyses (PPA) were performed for the primary outcome, including only satisfying protocol treatment and therefore solely subjects who completed at least six of the training modules.

#### Response analyses

To examine the clinical significance of a positive outcome, the reliable change index (RCI) defined by Jacobson and Truax was used [90]. In this manner, the SD of the norm population (6.2) and the reliability of the PSS-10 scale (α = .91) from samples in 2006 and 2009 were used in the respective formula [1.96*SD1*sqrt(2)*sqrt(1-rel)]. Eventually, an individual change over time was considered significantly reliable when the PSS-score differed more than ±5.16 points from T1 to T2 and T1 to T3. A cut-off value for symptom-free status was defined as more than 2 SDs below the mean (T1) value of the primary outcome (PSS-10 < 17.33). The number needed to treat (NNT) was calculated which indicates the average number of participants that need to be treated to achieve an additional response compared to the control condition and therefore prevent a non-response.

#### Mediation analysis

To explore how an effective stress reduction could lead to a prevention of depression, a mediation analysis based on a framework considering multiple pathways was conducted. Since depression can be preceded by stress, this was considered as potentially health impairing process as opposed to a health promoting process with resilience as a protective factor against the development or maintenance of depressive symptoms. Within this framework, perceived stress measured with PSS-10 [57, 69] was considered as potential risk factor to depression, hence to instigate a health impairing process. Resilience assessed with CD-RISC [55] on the other hand was assumed to be protective against the development of depressive symptoms as a result of work stress. A dual pathway with both factors mediating the efficacy in concert was also examined. For this purpose, a parallel mediation analysis was carried out using the PROCESS macro (v3.3) for SPSS (model 4) [91]. Temporal precedence was established by using T2-measurements of the mediators and the outcome values assessed at T3. In this model, baseline score for the potential mediators and the outcome were included as covariates [92]. Bias-corrected bootstrap with 10,000 samples was applied for indirect effects that were considered significant if 95% confidence intervals (CI) did not cover zero [91].

## Results

### Participants

**Fig. 1:**
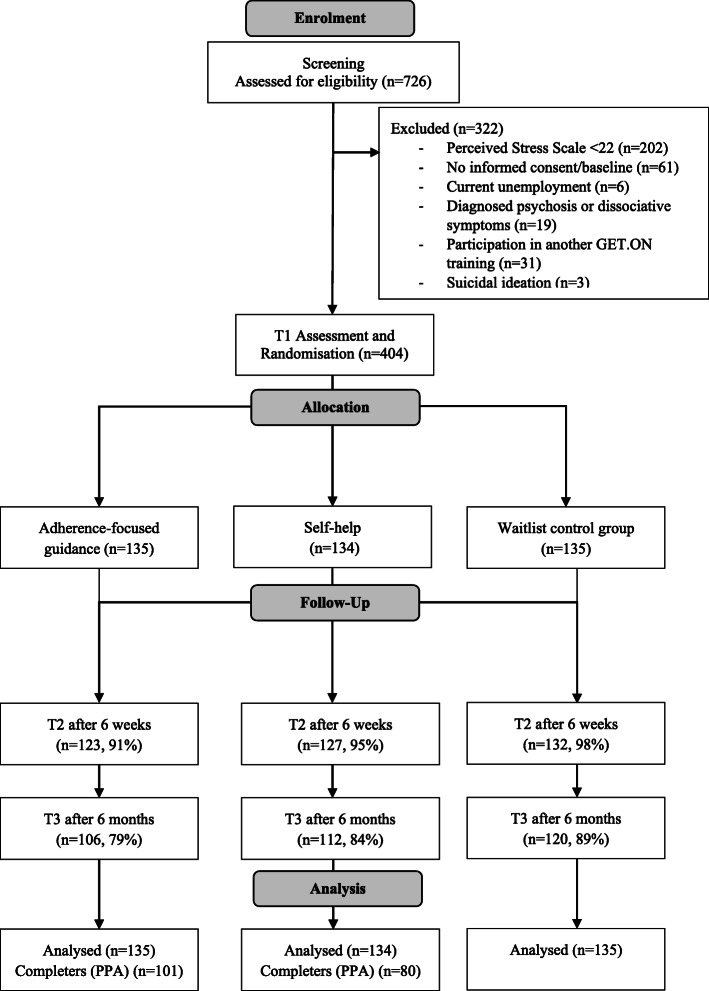
Study Flow

The sample consisted of 404 participants (77% female), aged 18–62 years (M = 41.93, SD = 9.14) that were randomly allocated to the three study conditions AFG (*n* = 135), SH (*n* = 134) or WLC (*n* = 135). At enrolment, PSS-10 mean scores numbered in 26.44 for AFG (SD = 2.92), 26.82 for SH (SD = 3.44), and 26.36 for WLC (SD = 3.05). All assessed baseline characteristics are summarised in Table 2 and the study flow is depicted in Fig. 1. The study aimed to include 408 subjects at T1 for allocation but with four participants failing to fully meet employment requirement, they had to be excluded from the analysis at closer data inspection. At enrolment, 726 subjects were recruited of which 322 did not fulfil the inclusion criteria. Satisfying protocol treatment for PPA analysis (completion of at least six modules) was available for 101 subjects in AFG (75%) and 80 in SH (60%). Study dropout rates for the assessments were as follows: at T2, AFG *n* = 12 (9%), SH *n* = 7 (5%), WLC *n* = 3 (2%) and at T3, AFG *n* = 29 (21%), SH *n* = 22 (16%), WLC *n* = 15 (11%). For the primary outcome, data were missing for 5% at T2 and 16% at T3. To examine if data was missing completely at random (MCAR) Little’s test was applied. The null hypothesis for Little’s test is that data are MCAR, thus patterns of missing scores are not associated with observed and unobserved factors among the subjects’ values. Little’s test of randomness failed statistical significance (*P* = 0.16), indicating the hypothesis of data MCAR did not need to be rejected. Therefore, multiple imputations were conducted [86].

**Table 2:** Baseline characteristics

Characteristics	AFG (***n*** = 135)	SH (***n*** = 134)	WLC (***n*** = 135)
Socio-demographic			
Age, mean (SD)	42.68 (9.09)	41.88 (9.01)	41.47 (9.22)
Women, n (%)	109 (80.70)	103 (76.90)	100 (74.10)
Marital status, n (%)			
Single	44 (32.60)	39 (29.10)	43 (31.90)
Married	65 (48.10)	65 (48.50)	63 (46.70)
Cohabited	14 (10.40)	14 (10.40)	15 (11.10)
Divorced	10 (7.40)	12 (9.00)	13 (9.60)
Widowed	2 (1.50)	4 (3.00)	1 (0.70)
Ethnicity, n (%)			
Caucasian/white	102 (75.60)	110 (82.10)	116 (85.90)
Asian	/	1 (0.70)	/
Hispanic	2 (1.50)	1 (0.70)	/
Prefer not to say	31 (23.00)	22 (16.40)	19 (14.10)
Educational level, n (%)			
Low	5 (3.70)	3 (2.20)	4 (3.00)
Middle	43 (31.90)	38 (28.40)	25 (18.50)
High	87 (64.40)	93 (69.40)	106 (78.50)
Employment			
Full-time, n (%)	107 (79.30)	93 (69.40)	106 (78.50)
Part-time, n (%)	26 (19.20)	39 (29.10)	26 (19.30)
Sick leave, n (%)	2 (1.50)	2 (1.50)	3 (2.20)
Managerial position, n (%)	53 (39.30)	48 (35.80)	56 (41.50)
Work experience in years, mean (SD)	17.87 (9.79)	17.29 (10.42)	17.76 (10.57)
Work sectors, n (%)			
Service	28 (20.70)	27 (20.10)	29 (21.50)
Economy	24 (17.80)	24 (17.90)	27 (20.00)
Health	13 (9.60)	21 (15.70)	18 (13.30)
Social	22 (16.30)	26 (19.40)	26 (19.30)
Information technologies	19 (14.10)	14 (10.50)	13 (9.60)
Other	29 (21.50)	22 (16.40)	22 (16.20)
Income, n (%)			
Low	45 (33.30)	37 (27.60)	43 (31.90)
Middle	27 (20.00)	35 (26.10)	19 (14.10)
High	51 (37.80)	52 (38.80)	60 (44.40)
Use of health services			
Previous or current psychotherapy, n (%)	62 (45.90)	71 (53.00)	72 (53.30)
Previous health training, n (%)	18 (13.30)	9 (6.70)	20 (14.80)

### Primary outcome analysis

**Fig. 2:**
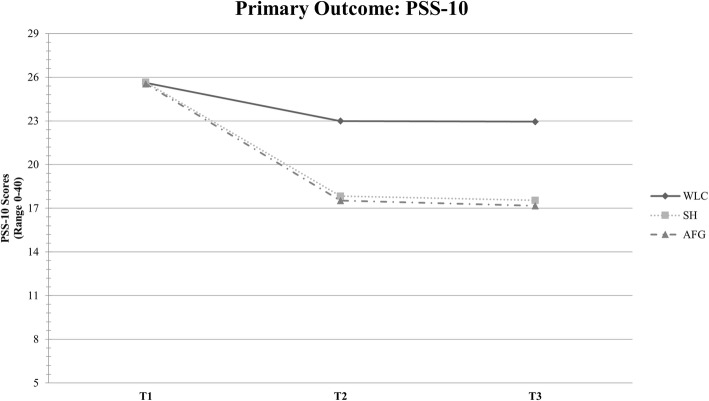
Comparison of adherence-focused guidance (AFG), self-help (SH) and waitlist control condition (WLC) on development of perceived stress measured with PSS-10 at T1 (baseline), T2 (after 7 weeks) and T3 (6-month follow-up)

All three study groups showed an improvement in perceived stress levels from T1 to T2 and T3 (Fig. 2). At T2, analysis of covariance according to the study protocol [35] revealed a significant group effect (F_2,400_ = 36.08, *P* < .001) with large between group effect sizes for both AFG (d = 0.83; 95% CI 0.58–1.08) and SH (d = 0.88; 95% CI 0.63–1.13) in comparison to the control group (Table 3). A significant between group effect was also found for T3 (F_2,400_ = 37.04, *P* < .001) with large effect sizes for AFG (d = 0.85; 95% CI 0.60–1.10) and SH (d = 0.91; 95% CI 0.66–1.16) compared to WLC. No significant group differences were found between AFG and SH for the primary outcome. See Table 4 for means and standard deviations for all study groups on primary and secondary outcomes.

**Table 3:** Results of the ANCOVAs and Cohen’s d based on respective imputed means and standard deviations for all study groups on the primary and secondary outcomes

	T2^a^ between-groups effect			T3^a^ between-groups effect		
Outcome	d (95% CI)		ANCOVA	d (95% CI)		ANCOVA
	AFG vs. WLC	SH vs. WLC	F_(400,2)_	AFG vs. WLC	SH vs. WLC	F_(400,2)_
**Primary Outcome**						
PSS-10	0.83 (0.58–1.08)	0.88 (0.63–1.13)	36.08***	0.85 (0.60–1.10)	0.91 (0.66–1.16)	37.04***
PSS-10 (PPA)^b^	1.00 (0.61–1.38)	1.11 (0.71–1.52)	19.75***	1.22 (0.83–1.62)	1.10 (0.70–1.50)	23.88***
**Secondary Outcomes**						
CES-D	0.74 (0.50–0.99)	0.62 (0.37–0.86)	28.64***	0.61 (0.37–0.85)	0.68 (0.43–0.92)	23.6***
CD-RISC	0.69 (0.44–0.94)	0.54 (0.29–0.78)	27.82***	0.54 (0.30–0.78)	0.60 (0.35–0.84)	19.71***
ERI (effort)	0.21 (0.03–0.45)	0.19 (0.05–0.43)	2.72	0.29 (0.05–0.53)	0.40 (0.16–0.64)	5.87**
ERI (reward)	0.23 (0.01–0.70)	0.19 (0.05–0.43)	6.93**	0.13 (0.11–0.37)	0.25 (0.01–0.49)	4.23*
MBI-GS-D	0.80 (0.55–1.05)	0.83 (0.58–1.08)	31.83***	0.84 (0.59–1.09)	0.91 (0.66–1.17)	34.73***
UWES (vigor)	0.36 (0.12–0.60)	0.31 (0.07–0.55)	9.52***	0.33 (0.09–0.57)	0.42 (0.18–0.66)	12.66***
UWES (dedication)	0.30 (0.06–0.54)	0.14 (0.10–0.38)	7.62**	0.16 (0.07–0.40)	0.20 (0.04–0.44)	4.69**
UWES (absorption)	0.26 (0.02–0.50)	0.18 (0.06–0.42)	4.61*	0.31 (0.07–0.55)	0.28 (0.04–0.52)	6.12**
WLQ (presenteeism)	0.27 (0.04–0.51)	0.46 (0.22–0.70)	5.45**	0.71 (0.46–0.95)	0.69 (0.44–0.93)	20.47***
WAI	0.40 (0.16–0.64)	0.43 (0.19–0.67)	7.51**	0.42 (0.17–0.66)	0.57 (0.33–0.82)	11.61***

*Abbreviations*: *PSS-10* Perceived Stress Scale, *PPA* Per protocol analysis, *CES-D* Centre for Epidemiological Studies’ Depression Scale, *CD-RISC* Connor-Davidson-Resilience scale, *ERI* Effort-reward imbalance, *MBI-GS-D* Maslach Burnout Inventory, *UWES* Utrecht Work Engagement Scale, *WLQ* Work limitations questionnaire, *WAI* Work ability index

Bonferroni-adjusted significance levels used *p < 0.017; **p < 0.003; ***p < 0.0003

^a^Missing data handled by multiple imputation

^b^F_(217,2)_

**Table 4:** Means and standard deviations for all study groups on primary and secondary outcomes

	T1 (baseline)						T2 (7 weeks post-baseline)^﻿a^						T3 (6 months post-baseline)a					
	AFG		SH		SH		AFG		SH		WDC		AFG		SH		WLC	
	M	SD	M	SD	M	SD	M	SD	M	SD	M	SD	M	SD	M	SD	M	SD
**Primary Outcome**																		
PSS-10	25.54	4.39	25.65	4.05	25.60	3.97	17.52	5.91	17.83	5.90	22.98	6.48	17.17	6.18	17.54	6.31	22.95	6.43
PSS-10 (PPA)	25.88	4.36	25.73	4.03	25.63	3.58	17.04	6.00	16.48	5.76	23.20	6.50	16.69	6.18	16.73	7.13	24.35	6.34
**Secondary Outcomes**																		
CES-D	19.28	7.21	18.44	6.63	18.59	6.63	11.90	6.92	12.65	7.60	17.58	8.27	12.61	7.39	12.08	7.41	17.38	8.20
CD-RISC	20.01	6.13	19.94	5.92	19.49	6.40	24.00	6.20	22.96	6.04	19.49	6.82	23.54	6.63	23.89	6.54	19.99	6.49
ERI (effort)	10.80	1.45	10.49	1.60	10.76	1.37	9.64	1.73	9.67	1.84	10.02	1.88	9.60	1.89	9.33	2.15	10.16	2.00
ERI (reward)	16.39	4.13	16.72	3.76	16.82	3.65	17.42	3.90	17.23	3.70	16.53	3.79	17.42	3.71	17.90	3.87	16.93	3.83
MBI-GS-D	4.73	0.82	4.70	0.76	4.84	0.67	3.95	0.92	3.92	0.92	4.67	0.89	3.81	0.99	3.73	0.99	4.60	0.89
UWES (vigor)	2.83	1.26	2.83	1.25	2.84	1.28	3.13	1.24	3.08	1.24	2.67	1.33	3.03	1.25	3.14	1.23	2.60	1.32
UWES (dedication)	3.24	1.46	3.16	1.48	3.28	1.45	3.59	1.34	3.37	1.41	3.17	1.50	3.26	1.37	3.32	1.41	3.03	1.42
UWES (absorption)	3.09	1.49	3.04	1.50	3.06	1.55	3.31	1.36	3.21	1.48	2.94	1.50	3.17	1.35	3.13	1.41	2.74	1.42
WLQ (presenteeism)	5.12	2.61	4.97	2.56	5.55	2.51	4.17	2.42	3.71	2.48	4.85	2.46	3.26	2.33	3.24	2.51	4.93	2.40
WAI	5.86	1.99	5.78	1.83	5.57	2.04	6.68	1.98	6.72	1.86	5.88	2.05	7.01	2.00	7.31	1.93	6.19	1.94

Abbreviations: *PSS-10* Perceived Stress Scale; *PPA* Per protocol analysis; *CES-D* Centre for Epidemiological Studies’ Depression Scale;

*CD-RISC* Connor-Davidson-Resilience scale; *ERI* Effort-reward imbalance; *MBI-GS-D* Maslach Burnout Inventory; *UWES* Utrecht Work Engagement Scale; *WLQ* Work limitations questionnaire; *WAI* Work ability index

a = Missing data handled by multiple imputation

#### Reliable change, symptom-free status, NNT

At T2, the majority of subjects in the intervention groups showed a reliable improvement on perceived stress assessed with PSS-10 (AFG: 84/123, 68%; SH: 80/127, 63%) compared to participants in WLC (39/131, 30%). Fewer symptom deterioration was found in the intervention groups (AFG: 3/123, 2%; SH: 3/127, 2%) than for WLC (7/131, 5%). These differences were statistically significant [χ^2^ = 4, (*N* = 381) = 45.11, *P* < .001].

At T3, a reliable improvement was present for both AFG (77/106, 73%) and SH (72/112, 64%) whereas this number was lower for WLC (26/119, 22%). Reliable deterioration again turned out lower for AFG (3/106, 3%) and SH (2/112, 2%) in comparison to WLC (7/119, 6%). Again, these differences were statistically significant [χ^2^ = 4, (*N* = 337) = 68.66, *P* < .001]. The NNT to achieve reliable improvement between T1 and T3 is 6, 95% CI [3.1, 26.7]. Furthermore, a significantly larger number of participants in the intervention groups could be considered as symptom-free (SH: 57, 43%; AFG: 69, 51%) compared to WLC (24, 18%) at T3 [χ^2^ = 2, (*N* = 404) = 34.64, *P* < .001].

#### Sensitivity analyses

In accordance with the study protocol [35], PPA including only subjects’ satisfying protocol treatment were conducted to test for the robustness of the main analyses (ITT) and revealed similar results. Significant between group effects were observed at T2 (F_2,217_ = 19.75, *P* < .001) with large effect sizes for AFG (d = 1.00, 95% CI 0.61–1.38) and SH (d = 1.11, 95% CI 0.71–1.52) compared to WLC. At T3, a significant group effect was found (F_2,217_ = 23.88, *P* < .001), again with large effect sizes for AFG (d = 1.22; 95% CI 0.83–1.62) and SH (d = 1.10; 95% CI 0.70–1.50). Again, the guided (AFG) and unguided (SH) conditions did not differ significantly from each other.

In addition, sensitivity analyses were performed to prevent bias due to the choice of the statistical approach. Therefore, all hypotheses were also tested applying repeated measures analysis of variance (ANOVA). All results could be replicated as indicated by the respective time x group effect.

### Secondary outcome analyses

Results of the ITT-analyses for the secondary outcomes are shown in Table 3. For both T2 and T3, ANCOVAs resulted in highly significant between-group effects with significance levels of *P* < .001 for most of the outcomes. The only non-significant result was the between-group effect assessed for ERI-effort at T2 (*P* = 0.67). At T2, effect sizes ranged from small (e.g., ERI-reward with d = 0.21 for AFG and d = 0.19 for SH) to moderate (e.g., WAI with d = 0.40 for AFG and d = 0.43 for SH) to large (e.g., MBI-GS-D with d = 0.80 for AFG and d = 0.83 for SH). For T3, detected changes in the effect sizes were mixed with partly decreases of already low effect sizes (e.g., ERI-reward with d = 0.13 for AFG and d = 0.25 for SH) and partly enhancements of large effect sizes (e.g., MBI-GS-D with d = 0.84 for AFG and d = 0.91 for SH).

### Training satisfaction and intervention usage

**Fig. 3:**
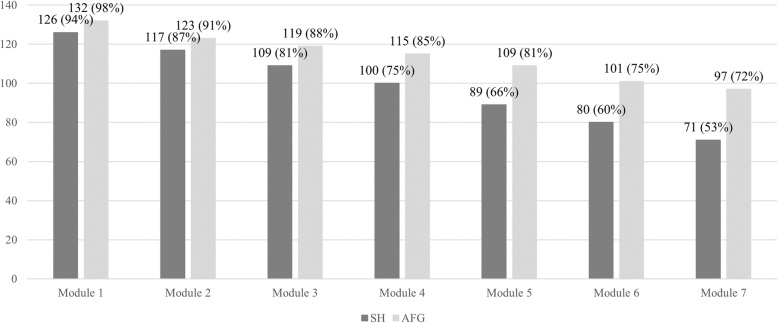
Number (percentages) of participants who completed the module (based on log-data)

Client satisfaction assessed with CSQ-I did not significantly differ for the study arms, neither at T2 (F_267,1_ = 0.61, *P* = .437), nor at T3 (F_267,1_ = 0.47, *P* = .494). Frequencies for module completion differed significantly from each other (F_267,1_ = 35.31, *P* = .008). On average, subjects in the AFG group completed 5.9 modules (*SD* = 2.01) compared to 5.17 in the SH condition (*SD* = 2.38). As shown in Fig. 3, the majority in AFG completed the seven core modules (97/135, 72%) compared to SH (71/135, 53%).

### Frequencies of given feedback and reminders

Table 5 shows the frequency distribution of responses to the questionnaire used to gather feedback on the provided guidance format. In the AFG condition, 17 subjects stated they have made use of feedback on demand. However, the manual check for these numbers on the messaging platform showed that 37 participants attempted to establish contact to an e-coach. 60 feedbacks were inquired in total across all participants (per subject: *M* = 0.44, *SD* = 0.94, range 0–5). Additionally, 453 reminders were sent (per subject: *M* = 3.36, *SD* = 1.88, range 0–9). Hence, e-coaches spent most of the time rather on adherence monitoring than giving feedback.

**Table 5:** Frequencies of answers to the guidance format questionnaire

	AFG (*n* = 134)^a^	SH (*n* = 135)^a^
**1 Which study group were you assigned to?**		
AFG	51 (38%)	6 (4%)
SH	71 (53%)	119 (88%)
**2 Did you make use of feedback on demand?**		
Yes	17 (13%)	n/a
No	39 (29%)	7 (5%)
**3 Would you have preferred regular feedback by an e-coach on your assignments?**		
Yes	58 (43%)	58 (43%)
No	39 (29%)	31 (23%)
No preference	25 (19%)	36 (27%)
**4 Which guidance format would you prefer if you would participate in a similar training again?**		
Regular feedback by an e-coach	64 (48%)	56 (41%)
Feedback-on-demand	47 (35%)	48 (36%)
Self-help (no feedback) and only technical support	11 (8%)	21 (16%)

^a^Percentages in relation to total samples

### Mediation analysis

**Fig. 4:**
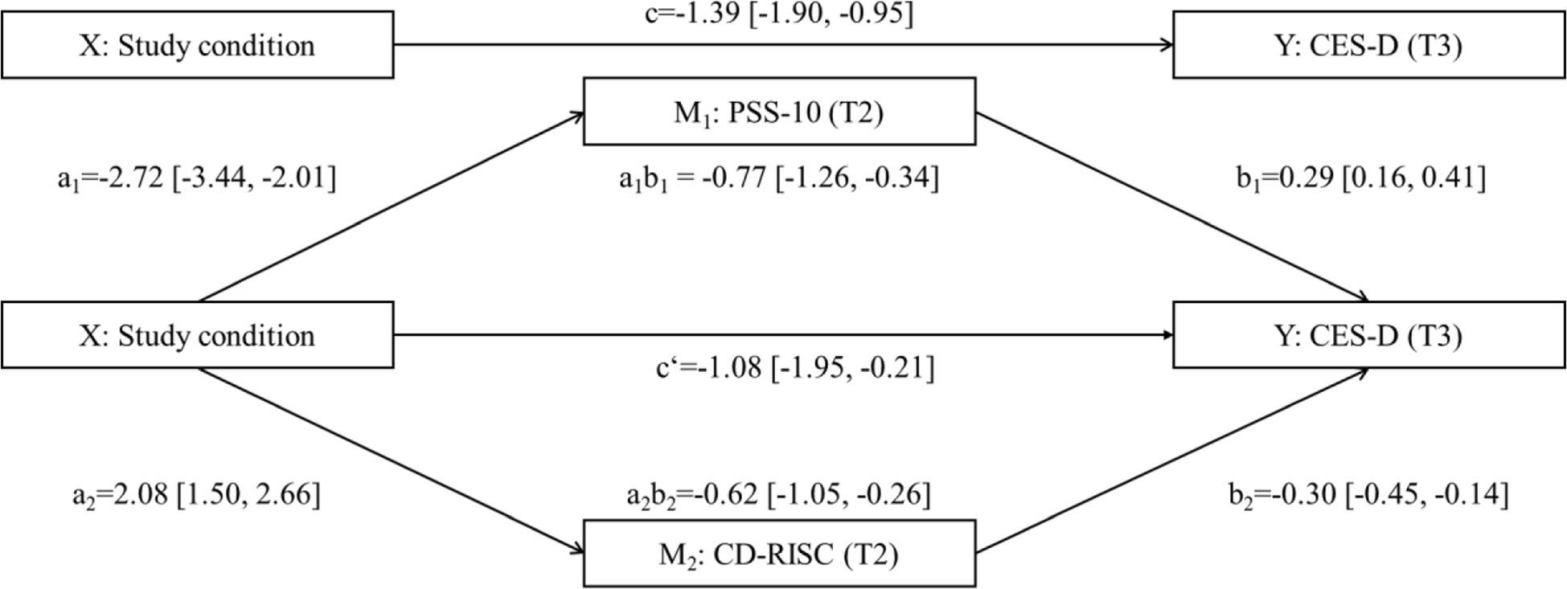
Parallel multiple mediation analysis with study condition as independent variable (coded 0 = waitlist control group, 1 = self-help, 2 = adherence-focused guidance), perceived stress (PSS-10) and resilience (CD-RISC) scores at post-intervention (T2) as mediators, 6-month follow-up depression values (T3) as outcome variable, and baseline values of mediators and outcome as covariates. Unstandardised beta coefficients are shown with 95% (bootstrapped biased corrected) CIs in parentheses

As depicted in Fig. 4, perceived stress assessed with PSS-10 at T2 significantly mediated the intervention effect on depression (CES-D) at T3: a_1_b_1_ = − 0.77, 95% CI [− 1.26, − 0.34]. Resilience measured with CD-RISC at T2 also significantly mediated the intervention effect on depression (CES-D) at T3: a_2_b_2_ = − 0.62, 95% CI [− 1.05, − 0.26]. After these mediators were incorporated into the model, the direct effect of the intervention on depression remained significant: c’ = − 1.08 [− 1.95, − 0.21].

## Discussion

This study aimed to investigate the comparative efficacy of different guidance formats on perceived stress within a web-based occupational SMI. Three arms were included in this trial to compare adherence-focused guidance, self-help and a wait list control group. With work stress being a major risk factor for the pathogenesis of depression which again is a leading cause for the contribution to the burden of disease [8], another objective was to reveal important insights into mechanisms of change to explore by which processes the SMI could have an impact on depressive symptoms. Understanding these mechanisms of change is of great interest and importance for health promotion, as offering SMI is a regarded strategy to prevent depression. To investigate potential mediators of the SMI’s efficacy on a reduction of depressive symptomatology, perceived stress was regarded as potentially health impairing mediator while resilience opposingly was considered a protective factor.

The results suggest that the intervention could effectively reduce perceived stress in employees, irrespective of the guidance format. Thus, the expected difference in efficacy between the two delivery modes AFG or SH was not found. For both intervention groups, the effect could be maintained at T3. Sensitivity analyses corroborated results of the ITT analyses and revealed even larger effect sizes.

The present study demonstrated higher effect sizes than a recent meta-analysis across 26 studies on web-based SMI with various guidance formats compared to different kinds of control groups (wait list control, attention control, alternative or no treatment) with an overall effect size of d = 0.43 for stress as primary outcome [15]. The yielded lower overall effect size compared to the higher effect sizes found in this study could be due to several reasons. It is important to note that heterogenous, not-specifically occupational interventions were included in this meta-analysis. Thus, study conditions, guidance formats, delivery mode or the length of these interventions could be various. This also accounts for the applied recruitment strategy which can vary between the studies and was shown to enhance treatment effects when open recruitment was used like in this trial [29]. The high levels of perceived stress subjects reported in this study at enrolment might indicate a high intrinsic motivation for the participation in this SMI and could have led to a greater benefit compared to subjects with lower baseline scores. Furthermore, higher levels of perceived stress at baseline might lead to a greater effect size due to the potential of improvement compared to lower scores at study enrolment.

Another potential explanation for the higher effect sizes found in this study might be found in the fact that the training comprises just two main components, namely emotion regulation and problem solving that are theoretically grounded in Lazarus’ transactional model of stress [60], comprising the two core components emotion regulation and problem solving. Research on traditional stress management programs suggests focussing on fewer components could be more effective than adding numerous modules [93]. This could possibly facilitate positive exercise effects and foster learning experiences due to the repeated revision of training contents. To the best of our knowledge, this is the first study on a web-based SMI investigating the comparative efficacy of different guidance formats within one trial, thus evidence for differential effects is limited and the present results can only be compared to heterogenous studies that each individually examined mostly only one type of guidance. Results of two individual trials investigating the same web-based SMI with each AFG [49] and SH [36] both also demonstrated comparably high effect sizes. In brief summary, this study is in line with previous research and adds to the hitherto missing evidence of the comparative efficacy of different guidance formats within one trial.

Concerning the non-significant differences between AFG and SH with regard to effects on the primary outcome, it should be noted that the intensity of guidance for AFG was per se low, and most resources were spent on adherence monitoring instead of providing feedback on demand. Albeit non-significant differences for the primary outcome, adherence in terms of completed modules was significantly higher for AFG than SH in this study. Another study investigated the comparative efficacy of AFG and SH under similar conditions within an Internet Intervention for problematic drinking in employees and also showed that despite missing between group differences, adherence was better for AFG [94]. Equally in the present trial, participants in the AFG condition completed significantly more modules (5.90) compared to SH (5.17). Additional to the monitoring, participants in the AFG condition could receive feedback on demand. 28% of the subjects in this group (37/134) made use of the latter and received 60 feedbacks in total, next to 453 reminders that were sent in sum. Similarly small numbers of participants demanding feedback were found before in trials on the same SMI, with e-coaches also spending more time on adherence monitoring [50]. In these trials, the amount of subjects making use of feedback on demand numbered 6% (8/132) [49] and 11% (15/102) [50]. In the aforementioned RCT on a web-based intervention for problematic drinking in employees, 10% (15/144) made use of feedback on demand [94].

At first glance, this could lead to the conclusion that guidance might not be conducive to the efficacy of web-based SMI. However, this needs to be considered cautiously. First, this would be a false conclusion since the utilisation rate of AFG was low, not only in the present trial but also previous ones [50, 94]. Second, reasons for this can be manifold. For example, results of the questionnaire on utilisation of and satisfaction with AFG indicated that the majority of this group was not even aware of their assigned condition. Instead, more than half of the AFG-subjects (53%) assumed receiving a pure SH-intervention. Moreover, 43% of the AFG-subjects (SH: 43%) stated they would have preferred regular feedback by an e-coach on their assignments which would furthermore be preferred by 48% (SH: 41%) if they would participate in a similar training again. In conclusion, it seems that the low utilisation rate of feedback on demand as part of AFG could possibly be due to a lack of awareness of what was offered. It can be speculated that information on the availability of personal feedback from the e-coach should have been presented more prominently and that such awareness-raising measures could have been helpful. In this way, e-coaches could for example contact participants more actively by encouraging them to make us of the AFG and offering their feedback. Another possibility to promote acceptance could be the use of educational videos to explain the delivery mode in more detail. To conclude, it seems that the monitoring component of AFG might promote adherence but might not be sufficient to enhance efficacy with such low utilisation rates of the offered feedback on demand. The superiority of AFG for adherence was not mirrored for the outcomes since results demonstrated high effect sizes for SH and adherence defined as the average amount of completed modules was satisfying for this group. On the one hand, this leads to the question if the usage of AFG is justifiable against the background of required resources. On the other hand, the level of guidance was per se low for AFG, thus differences compared to SH might be challenging to detect because they could be small by default.

Analyses of secondary measures demonstrated that the present web-based SMI came into effect on the majority of the outcomes such as depression, resilience, emotional exhaustion, work engagement, presenteeism and work ability. In more detail, effect sizes for the reduction of depressive symptoms were found to be high for each intervention group compared to WLC (at post-intervention, d = 0.62 for SH and d = 0.74 for AFG). These effect sizes are higher than those observed in a meta-analysis on the efficacy of SMI on depression (d = 0.34) [15]. Notably, earlier studies reported consistently higher effect sizes for guided Internet Interventions than unguided interventions [15, 39]. It seems interesting to note that all variables directly related to the subjects changed over time and that no significant effect was found for ERI-effort at T2 (*P* = .67) which from a conceptual point of view is a measure of the workplace situation. Comparable to the primary outcome, no significant differences were found between AFG and SH, although this should again be interpreted cautiously for already stated reasons.

Another important objective of this study was to examine mediating interventional effects on depressive symptoms. This is of great interest for practical and theoretical reasons and there is a growing need for such research [95, 96]. To date, research is limited to observational studies that identified stress as risk factor for the development of depression [6]. The use of SMI to prevent depression therefore seems reasonable, though interventional studies and more in-depth analyses of this relationship are missing yet. Beyond the reduction of perceived stress, the present SMI became efficacious on a pathological dimension by also reducing depressive symptoms. This result highlights the potential of the SMI in regard to the prevention of mental health disorders.

Results of the mediation analysis demonstrated the applicability of the proposed framework and showed that both considered paths mediated the intervention’s effect on depressive symptoms. On the one side, the intervention could positively affect perceived stress which again had a positive preventive effect on depressive symptoms. On the other side, the intervention also positively affected resilience which seemed conducive to a health promoting process and depressive symptoms eventually. These results reveal interesting insights into the question of how depressive symptoms could be reduced and prevented. Depressive symptomatology was less pronounced when participants on the one side perceived less stress and on the other side were more resilient. A similar pattern with interventional effects based on reducing negative factors on the one hand and building up positive ones on the other hand was also found in an earlier study on a web-based gratitude training which examined mediation analyses based on the same approach of a dual pathway [97].

Despite years of research on the effects of stress and resilience in an occupational context, mechanisms of change of SMI have not received attention yet. However, research not limited to interventional studies showed that resilience as a core protective factor can be developed at work and comes into effect especially in employees at a greater risk of experiencing high strain [98]. Taken together with the present results, important implications for practitioners and health intervention developers can be drawn. The present interventional study is in line with results of earlier observational studies and adds further evidence. Taken together, the findings suggest a contentual integration of both, the health impairing and health promoting processes. Participants should be supported in reducing risk factors such as stress and building up protective factors such as resilience.

Previous research has shown that Internet Interventions could be as effective as face-to-face settings [26–28]. However, evidence is only available on indirect comparisons; well designed and adequately powered head-to-head comparisons are yet missing [99]. Traditional face-to-face settings are characterised by certain features such as a group setting, a fixed venue or a pace depending on the training schedule which obviously are different to the typical characteristics of Internet Interventions (see [99] for a full comparison of both). The differences in the typical characteristics of both delivery modes can be an advantage for one participant (e.g. experiencing a motivating group atmosphere) and an obstacle for the other (e.g. high threshold to participate in group setting with social anxiety). Unfortunately, evidence for selective indication is missing. Interestingly, several studies on web-based SMI reported that the vast majority of participants have no experiences with previous face-to-face trainings [36, 49, 61], indicating that both delivery modes (traditional vs. online) reach their audience and thereby increase the overall reach and uptake of SMI.

This study had several limitations. First, the utilised SMI was for the indicated prevention of work stress related adverse health outcomes. This required an elevated stress level for participants to be included in the study. Therefore, resulting conclusions from this study cannot be generalised to populations showing lower stress levels or to a setting in universal prevention. However, from a methodological perspective, this allows for comparisons to studies with similar inclusion criteria. Second, limited generalisability of the results due to the recruitment strategy in this trial must be considered. This trial directly addressed subjects and therefore provides no insights with regard to an implementation into Corporate Health Management. In future research, it should therefore be considered offering SMI for employees in actual occupational setting, e.g. company health management programs. Third, only few participants in the AFG condition made use of the personalised feedback and many of them were not aware that their guidance comprised this opportunity. The low utilisation rate of the guidance suggests it would have been advisable to conduct a pre-test. This could ensure that participants who do not engage with the offered components of guidance (i.e. feedback on demand) deliberately decide against this but are aware of their options.

Finally, further research is needed to fully capture the comparative efficacy of different guidance formats. Participants should receive guidance facilitating and awareness-raising measures to know that and how they can make use of the accompanying guidance. Ideally, they could make an informed decision about the extent to which they would like to inquire feedbacks and determine the dose-response relationship. Other reasons for the low utilisation rate for AFG could also be considered and influencing factors examined, for example if and to what extent personal presence of e-coaches is necessary. Due to the low intensity of guidance for AFG, existing differences for the comparative efficacy could be difficult to detect compared to SH. Therefore, more heterogeneous trials on the impact of guidance are still necessary and could for example compare adherence-focused to content-focused guidance [38]. For future studies, it could also be an interesting approach to explore and disentangle the effects of the two single components of AFG, namely monitoring and giving feedback on demand.

## Conclusions

The present study contributes to the scarce evidence on the comparative impact of guidance to the efficacy of SMI, next to mechanisms of change of depressive symptoms. To the best of our knowledge, no previous study has compared different guidance formats within a single trial on SMI. The results showed that the SMI was effective in reducing perceived stress as primary outcome, next to various other work- and mental health-related outcomes such as depression or burnout. However, no between group differences were found for the effect sizes when comparing AFG to SH. This supports a growing body of research showing that SMI with a limited amount of resources can be effective. Despite missing significant between group differences for the outcomes, it should be noted that adherence in terms of module completion was significantly better for AFG than SH. Due to the low utilisation rate of AFG, the results should furthermore be interpreted cautiously regarding the question of how much guidance is necessary for an efficacious and well-accepted SMI. Future studies should therefore focus on further capturing differences between different guidance formats and explore how the better adherence for AFG could also lead to better outcomes compared to SH. Guidance formats could for example be more distinguishable than AFG compared to SH to examine the incremental value of more intensive guidance. Another important conclusion for future studies is to attempt higher utilisation rates when guidance is offered, irrespective of its kind. Furthermore, the results showed that stress and resilience mediated the effects on depressive symptomatology. This interventional study therefore adds evidence to earlier observational studies on the relationships between stress and depression and resilience and depression. Important implications for practitioners and research can be derived to consider both pathways of a health impairing and health promoting process in designing such interventions.

## Data Availability

The datasets used and/or analysed during the current study are available from the corresponding author on reasonable request.

## Abbreviations

AFG: Adherence-focused guidance
ANCOVA: Analysis of covariance
ANOVA: Analysis of variance
BDI: Beck depression inventory
CD-RISC: Connor-Davidson resilience scale
CES-D: Centre for epidemiological studies’ depression scale
CI: Confidence interval
CONSORT: Consolidated standards of reporting trials
CSQ-I: Client satisfaction questionnaire adapted to Internet-based interventions
ERI-SF: Effort reward imbalance questionnaire – short form
GCP: Good clinical practice
ITT: Intention-to-treat
JD-R: Job-demands-resources model
MBI-GS-D: Maslach burnout inventory
MCAR: Missing completely at random
NNT: Number needed to treat
PPA: Per protocol analysis
PSS-10: Perceived stress scale
RCI: Reliable change index
RCT: Randomised controlled trial
SD: Standard deviation
SH: Self-help
SMI: Stress management intervention
TAU: Treatment as usual
UWES: Utrecht work engagement scale
WAI: Work ability index
WHO: World health organization
WLC: Wait list control group
WLQ: Work limitations questionnaire
